# Time-Dependent Expression Profiles of microRNAs and mRNAs in Rat Milk Whey

**DOI:** 10.1371/journal.pone.0088843

**Published:** 2014-02-12

**Authors:** Hirohisa Izumi, Nobuyoshi Kosaka, Takashi Shimizu, Kazunori Sekine, Takahiro Ochiya, Mitsunori Takase

**Affiliations:** 1 Nutritional Science Institute, Morinaga Milk Industry Co., Ltd., Zama, Kanagawa, Japan; 2 Division of Molecular and Cellular Medicine, National Cancer Center Research Institute, Chuo-ku, Tokyo, Japan; Keio University, Japan

## Abstract

Functional RNAs, such as microRNA (miRNA) and mRNA, are present in milk, but their roles are unknown. To clarify the roles of milk RNAs, further studies using experimental animals such as rats are needed. However, it is unclear whether rat milk also contains functional RNAs and what their time dependent expression profiles are. Thus, we prepared total RNA from whey isolated from rat milk collected on days 2, 9, and 16 postpartum and analyzed using microarrays and quantitative PCR. The concentration of RNA in colostrum whey (day 2) was markedly higher than that in mature milk whey (days 9 and 16). Microarray analysis detected 161 miRNAs and 10,948 mRNA transcripts. Most of the miRNAs and mRNA transcripts were common to all tested milks. Finally, we selected some immune- and development-related miRNAs and mRNAs, and analysed them by quantitative PCR (in equal sample volumes) to determine their time-dependent changes in expression in detail. Some were significantly more highly expressed in colostrum whey than in mature milk whey, but some were expressed equally. And mRNA expression levels of some cytokines and hormones did not reflect the protein levels. It is still unknown whether RNAs in milk play biological roles in neonates. However, our data will help guide future *in vivo* studies using experimental animals such as rats.

## Introduction

Milk, the only nutritional source for newborn mammals, it contains many biologically active components, such as cytokines, growth factors, and hormones, which can modulate neonatal development [1]–[3]. In addition to these components, our group [4]–[6] and other groups [7]–[13] recently reported that human, bovine, and sow milks contain microRNAs (miRNAs), notably immune-related miRNAs. Moreover, we detected some milk protein-related and milk-derived exosome-related protein-encoding mRNAs in bovine milk using quantitative PCR (qPCR) [4]. Some of these reports also found that RNAs in milk are resistant to acidic conditions and to RNase [4], [6]. This suggests that RNAs in milk might be packaged into exosomes and/or microvesicles and may be able to function in the gastrointestinal tracts of neonates. However, it remains unclear whether RNAs in milk can function in living bodies. To clarify the roles of milk RNAs, further studies using experimental animals such as mice and rats are needed, but it is unclear whether rodent milk also contains these functional RNAs. According to previous studies [6], [12], the miRNA profiles of mature human breast milk differ between individuals, rather than according to milk collection time during the lactation period. These individual specificities might be due to genetic background/race and lifestyle/diet. Even in cows, which show less diversity than do humans, miRNA profiles are different between colostrum and mature milk [4], [7]. These differences suggest that each miRNA's role in neonates could be different at the postpartum stage. In experimental animal studies, it could be possible to minimize individual specificities and to investigate in detail using the same strain, diet, and precisely term-determined milk.

Milk consists of cells, fat, casein and whey. Several studies have reported that cells [14], the fat fraction [10], [11], [15], and whey [4]–[9], [12], [13] contain RNA. In this study, we focused on whey and examined exploratory. Whey contains many biological factors such as cytokines, hormones, immunoglobulins, exosomes and microvesicles, but there have been no comprehensive report of the milk whey mRNAs of any species, although mRNAs in human milk fat globules and bovine milk somatic cells have been investigated using microarray [10] and next-generation sequencing [14], respectively. We found that rat milk whey also contains functional RNAs and clarified the time-dependent expression changes in these functional RNAs during lactation. This paper provides basic information that will guide future studies of the roles of RNAs in milk whey.

## Materials and Methods

### Rats

All animal experiments were approved by the Institutional Animal Care and Use Committee of Morinaga Milk Industry Co., Ltd. and were performed in accordance with the Guide for the Care and Use of Laboratory Animals of Morinaga Milk Industry Co., Ltd.; with the Law Concerning the Protection and Control of Animals (Law No. 105; October 1, 1973; revised on June 22, 2005; revised Law No. 68); and with Standards Relating to the Care and Management of Laboratory Animals and Relief of Pain (Notification No. 88 of the Ministry of the Environment, Japan; April 28, 2006). Pregnant F344/N rats (14 days pregnant, 14 weeks old) were obtained from Japan SLC Inc. (Shizuoka, Japan). They were housed individually in cages under temperature controlled conditions (23±2°C) with a 12/12-h light–dark cycle, and were allowed free access to a commercial diet (MRSTOCK, Nosan Corporation, Kanagawa, Japan) and tap water.

### Milk and serum sample preparation

Milk was sampled on days 2, 9, and 16 postpartum according to the method of Keen [16] with slight modifications. Briefly, dams were separated from their litters for 3–4 h before milking and were milked for 30 min between 13:00 and 15:00. Oxytocin (1 I.U., Sigma-Aldrich, St. Louis, MO) was injected intraperitoneally 15 min before milking. The dams were lightly anesthetized with sevoflurane (Mylan Inc., Canonsburg, PA) and were milked by gentle hand stripping. Collected milk samples were immediately incubated at 4°C and were centrifuged (1,200×*g*, 4°C, 10 min) to remove fat, cells, and large debris. Defatted and cell eliminated supernatants were centrifuged (21,500×*g*, 4°C; 30 min twice, and 1 h) to remove residual fat and casein, yielding a clear supernatant (whey). Blood samples were obtained at day (d) 21 postpartum (the weaning period in rats) for analyzing serum RNA as representative body fluid RNA. Dams were sacrificed by deep anesthesia with sevoflurane and blood was collected from the inferior vena cava. Blood samples were centrifuged (1,200×*g*, 25°C, 10 min) to obtain serum. Whey and serum samples were passed through 0.65-µm, 0.45-µm, and 0.22-µm filters to remove residual cell debris and kept at −80°C until analysis.

### Total RNA extraction

Total RNA was extracted from whey and serum samples and purified using an miRNeasy Mini Kit (Qiagen, Hilden, Germany) as described previously [4]–[6]. Briefly, whey and serum samples were diluted in five volumes of QIAzol Lysis Reagent, mixed thoroughly by vortexing, and incubated for 5 min at room temperature (RT, 25°C) (denatured samples). Then chloroform (an equal volume to the sample volume) was added to the homogenates, which were mixed thoroughly by vortexing and incubated for 3 min at RT. The homogenates were centrifuged (12,000×*g*, 4°C, 15 min). The resulting aqueous phase was mixed thoroughly with 1.5 volumes of 100% molecular biology-grade ethanol and passed through an miRNeasy column. The column was washed according to the manufacture's protocol, and RNA was eluted in nuclease free water. The quantity and integrity of the RNA were assessed using an RNA 6000 Pico Kit, and the miRNA/small RNA ratio (default settings: miRNA, 10–40 nt; small RNA, 0–270 nt) was examined on an Agilent 2100 Bioanalyzer using a Small RNA Kit (all from Agilent Technologies, Santa Clara, CA).

### miRNA microarray experiments

To detect the expression of miRNAs in rat whey and serum, 70 ng of total RNA were labeled with cyanine 3 and hybridized to Rat miRNA Microarray Rel. 14.0 arrays using an miRNA Complete Labeling Reagent and Hyb Kit (Agilent Technologies). The Rat miRNA Microarray Rel. 14.0 screens for the expression of 388 rat mature miRNAs from Sanger miRBase Rel.14.0 (www.mirbase.org). Signals were detected with an Agilent DNA Microarray Scanner, and the scanned images were analyzed using Feature Extraction Software (ver. 9.5.3.1).

### mRNA microarray experiments

Complementary RNA was generated from 100 ng total RNA (in the case of serum, 100 ng total RNA was obtained by mixing three samples) and labeled with cyanine 3 using a Low RNA Input Linear Amplification Kit (Agilent Technologies). Labeled cRNA was hybridized to Whole Rat Genome Oligo Microarrays (4×44K) using a Gene Expression Hybridization Kit (Agilent Technologies). The Whole Rat Genome Oligo Microarray (4×44K) contains 41,090 probes (not including control probes). Signals were detected with an Agilent DNA Microarray Scanner, and the scanned images were analyzed using Feature Extraction Software (ver. 9.5.3.1).

### Microarray data analysis

Raw data from Feature Extraction Software were exported to GeneSpring GX ver. 11.5.1 (Agilent Technologies). Quantile normalization was used to normalize data. Entities were considered present if at least one of three samples per group was judged to be “present”. Furthermore, in the case of mRNA microarray data, transcripts with raw signals >20 were considered to be present. Target mRNAs of specific miRNAs were predicted using the Web-based prediction tool TargetScan [17] (www.targetscan.org) and the biological roles of miRNA targets and mRNA transcripts were analyzed by Ingenuity Pathway Analysis (IPA) (Ingenuity Systems, Redwood City, CA). All microarray data were deposited in the National Center for Biotechnology Information (NCBI) Gene Expression Omnibus (GEO) and are accessible through GEO Series accession number GSE44114 (www.ncbi.nlm.nih.gov/geo).

### Quantification of miRNA by qPCR

Complementary DNA was generated using an miScript Reverse Transcription Kit (Qiagen). Briefly, total RNA from 5 µL whey or serum was polyadenylated using poly (A) polymerase and cDNA was generated with reverse transcriptase using tagged oligo-dT primers. Then the cDNA was diluted in nine volumes of nuclease-free water and subjected to qPCR on a 7500 Fast Real-Time PCR System (Applied Biosystems, Foster City, CA) using an miScript SYBR Green PCR Kit and an miScript Primer Assay (both from Qiagen). The following real-time PCR protocol was used: initial activation of HotStartTaq DNA Polymerase (95°C, 15 min); 40 to 50 cycles of denaturation (94°C, 15 s), annealing (55°C, 30 s), and extension (70°C, 34 s); and melting curve analysis. The miScript Primer Assays we used for the target mature miRNAs are listed in Table S1. The data were analyzed using 7500 Software version 2.0.4. (Applied Biosystems) with the fixed cycle threshold (Ct) setting (ΔRn = 0.02, where ΔRn is the fluorescence of the reporter dye minus the baseline) to assign baseline values and the threshold for Ct determination.

### Quantification of mRNA by qPCR

Complementary DNA for the quantification of mRNA was generated using a High Capacity RNA-to-cDNA Kit (Applied Biosystems). Briefly, total RNA from 5 µL whey or serum was used to generate cDNA with reverse transcriptase and a primer cocktail (oligo-dT and random primers). The cDNA was diluted in nine volumes of nuclease free water and then subjected to qPCR on a 7500 Fast Real-Time PCR System using TaqMan Fast Advanced Master Mix and TaqMan probes (both from Applied Biosystems). The following real-time PCR protocol was used: uracil-N-glycosylase incubation (50°C, 2 min); polymerase activation (95°C, 20 s); and 40 to 50 cycles of denaturation (95°C, 3 s) and annealing and extension (60°C, 30 s). The TaqMan probes used for the target mRNAs are listed in Table S2. The data were analyzed using 7500 Software ver. 2.0.4. (Applied Biosystems) with the fixed Ct setting (ΔRn = 0.2) to assign baseline values and the threshold for Ct determination.

### ELISA assays

Whey and serum transforming growth factor (TGF)-βs exist as latent forms. Therefore, samples needed to be activated by acid. Serum samples were activated with 1 N HCl (1/4 volume of serum) for 30 min at RT and were neutralized with 0.5 M HEPES/1.2 N NaOH (1/5 volume of serum) according to the manufacturer's protocol. Whey samples were activated as described previously [18]. Briefly, whey was activated with 1.2 N HCl (1/10 volume of whey) for 30 min at RT and then neutralized with 0.5 M HEPES/0.72 M NaOH (1/4 volume of whey). Whey and serum TGF-βs concentrations were determined by ELISAs (TGF-β_1_, MB100; TGF-β_2_, DB250; R&D Systems, Abingdon, UK). IGF-I and adiponectin concentrations in whey and serum samples were measured using ELISA kits (IGF-I: MG100, R&D Systems) (adiponectin: KRP0041, Invitrogen, Carlsbad, CA).

### Multiplex assays

Concentrations of VEGF-α, CCL5 (also known as regulated on activation, normal T cell expressed and secreted, RANTES) and CXCL1 (also known as Gro/KC) in whey and serum samples were measured using a Milliplex Rat Cytokine/Chemokine Panel (RCYTO-80K, Millipore, Billerica, MA). Insulin concentrations in whey and serum samples were measured using a Milliplex Rat Bone Panel 1 (RBN1-31K, Millipore). These panels were processed using a Bio-Plex Protein Array System and the data were analyzed with Bio-Plex Manager 6.0 software (both from Bio-Rad Laboratories, Hercules, CA).

### Statistical analyses

Values are expressed as the mean ± SEM. The significance of differences was determined using the Tukey-Kramer honestly significant difference (HSD) test for multiple comparisons (JMP software; SAS institute Inc., Cary, NC). A *P*-value of less than 0.05 was considered to indicate a significant difference.

## Results and Discussion

**Figure 1:**
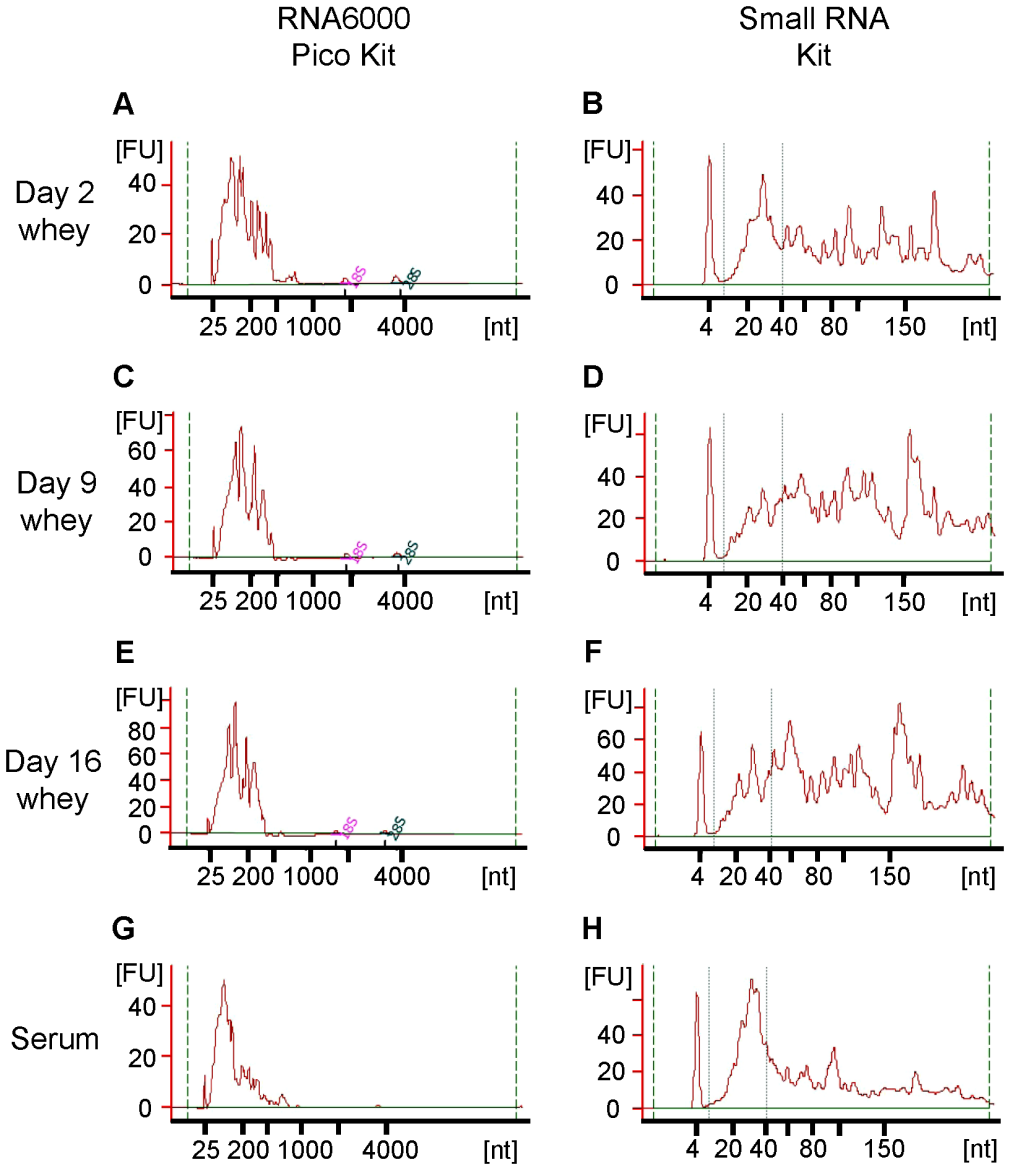
Bioanalyzer analysis of RNAs in rat whey and serum. (A, B) Day 2 whey; (C, D) Day 9 whey; (E, F) Day 16 whey; (G, H) Serum; (A, C, E, G) Analysis using the RNA 6000 Pico Kit; (B, D, F, H) Analysis using the Small RNA Kit. Whey RNA concentrations were very high (especially in day 2 whey), so diluted RNA results are shown in this figure. FU  =  fluorescence units.

**Figure 2:**
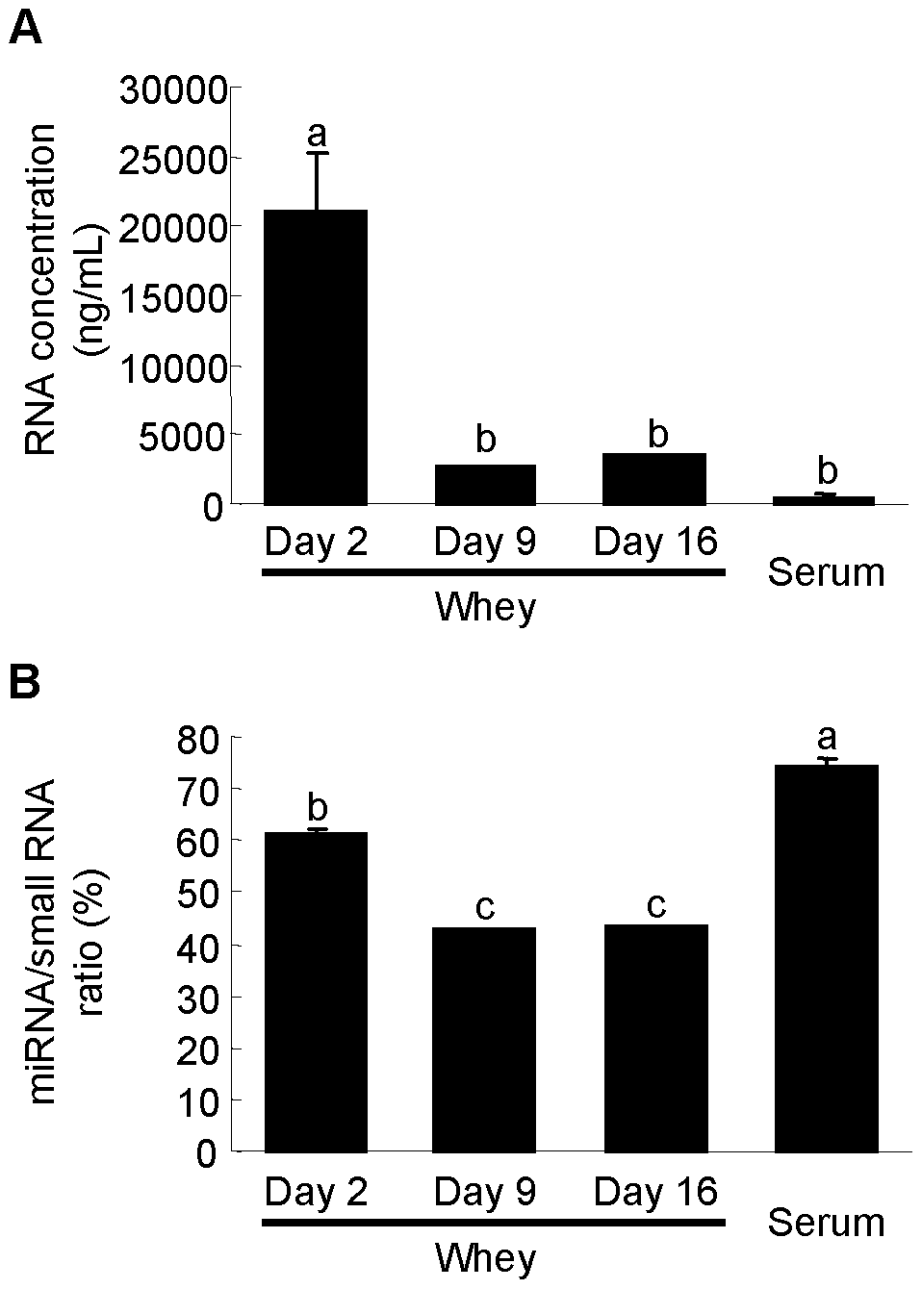
RNA concentration and miRNA/small RNA ratio of RNA samples purified from rat whey and serum. (A) RNA concentrations in rat milk whey and serum. (B) miRNA/small RNA ratio; Values are the mean ± SEM (n = 3). Means without letters in common differ (*P*<0.05, Tukey-Kramer HSD test).

**Figure 3:**
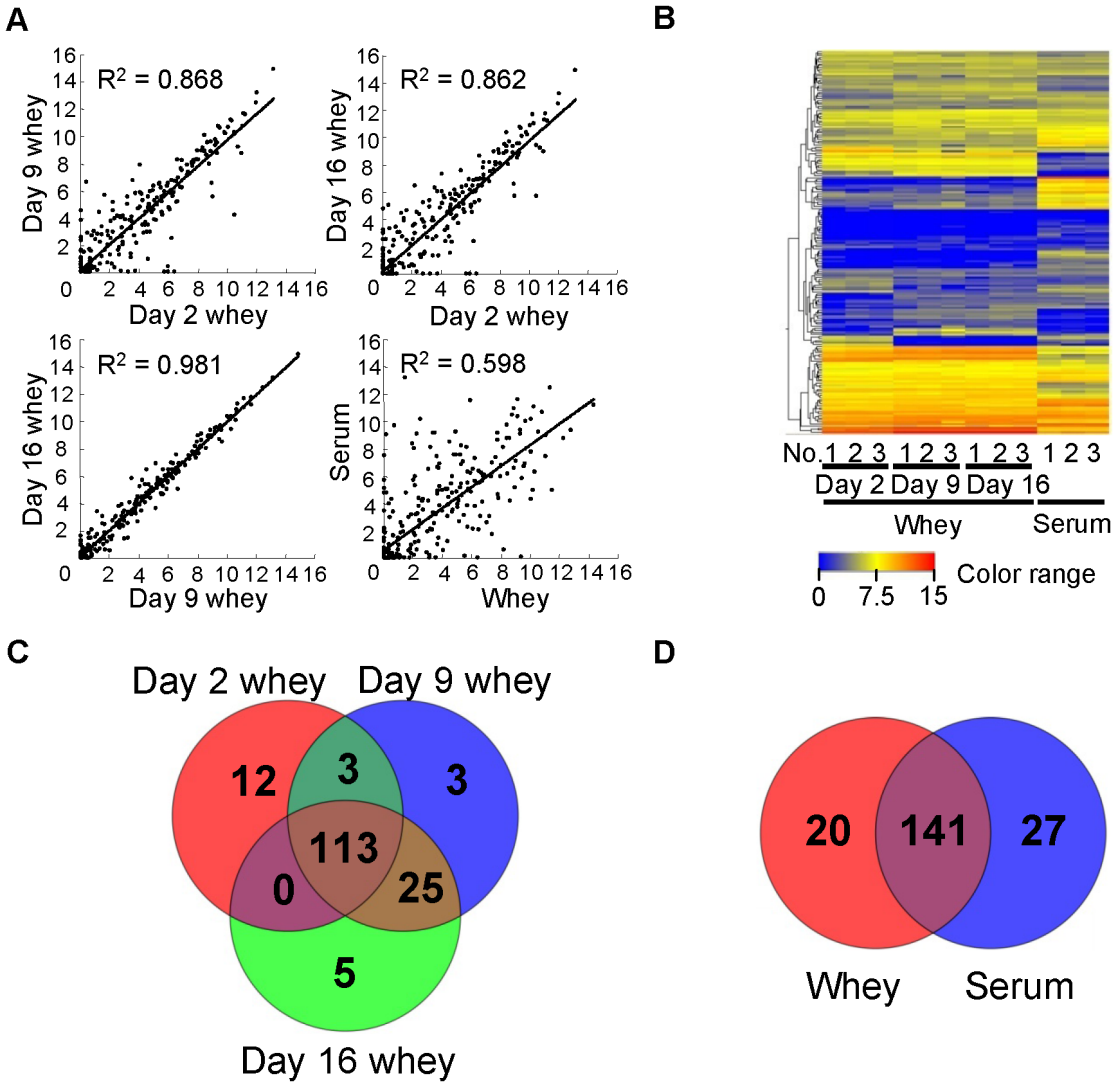
miRNA microarray profiles of rat whey and serum. (A) Scatter plots of averaged whey and serum array data. (B) Heat map of the normalized array data for miRNAs detected in whey or serum samples. (C) Venn diagram of numbers of miRNAs detected in each type of whey. (D) Venn diagram of numbers of miRNAs detected in whey and serum. Detailed signal intensities in heat map and miRNA species in Venn diagram are shown in Table S3.

We extracted total RNA from rat milk whey and serum, and analyzed it using a Bioanalyzer. There were no or very little ribosomal RNA (18S and 28S), but small RNAs (<300 nt) were present (Fig. 1). This result is in agreement with results for milk whey from other species [4]–[6], [8], [19], and suggests that the RNA in whey and serum is not derived from cells in milk and in blood, respectively. There have been few studies on RNA concentrations in milk. However, according to our previous studies, RNA concentrations in human milk whey range from ∼10 to several hundred ng/mL [6] and the concentration in bovine milk whey is several hundred ng/mL [4], [5]. In this study, we detected RNA in rat milk whey at concentrations in the range of several to tens of thousands of ng/mL (Fig. 2A). Milk is the only nutritional source for newborn mammals, and there are large differences in the nutritional components of different mammalian milks, which are adapted for the development of a specific type of offspring. Generally, if the development time is short, the milk is nutrient-dense [20]. Rats grow more quickly than humans and cows. Thus, the very high amount of RNA in rat milk whey compared to other species suggests that whey RNA may have some physiological roles in neonates. RNA concentrations were much higher in colostrum whey than in mature milk whey and serum (Fig. 2A), in agreement with results for bovine [4], [9]. In general, colostrum contains large amounts of immune-related components and is thus considered very important to neonates [2], [3]. This suggests that milk RNA may also have important roles in neonatal immune development. The miRNA/small RNA ratio was also higher in colostrum whey than in mature milk whey, but serum had the highest ratio (Fig. 2B). This shows that the RNA composition differs among colostrum whey, mature milk whey, and serum. A scatter plot analysis (Fig. 3A) of miRNA microarray data obtained using the same amount of total RNA (70 ng) showed that there are large differences between whey and serum, some differences between colostrum whey and mature milk whey, and few differences between mature milk whey in terms of miRNA expression patterns. We detected 161 of the 388 known rat miRNAs in whey. A total of 128 miRNAs were detected in colostrum (d 2) whey, 144 miRNAs in d 9 whey, and 143 miRNAs in d 16 whey (Fig. 3B–D). And 168 miRNAs were detected in serum (Fig. 3B, D). A total of 141 miRNAs were common to both whey and serum (Fig. 3D), but their signal intensities were quite different (Fig. 3B). Many miRNAs have been identified, but the targets and roles of very few of them have been experimentally confirmed. We used the Web-based miRNA target prediction tool TargetScan [17] to analyze the roles of the predicted targets by IPA. It was predicted that 11,634 mRNAs could be affected by the 161 miRNAs detected in whey. These predicted target mRNAs had roles such as “cellular development”, “cellular growth and proliferation”, and “tissue development” (Table 1).

**Table 1:** Functions of predicted targets of whey miRNAs.

Rank	Category	B-H *P*-value
1	Cellular Development	1.61E-62 - 4.57E-13
2	Cancer	1.12E-61 - 9.18E-13
3	Cell Cycle	1.28E-59 - 8.79E-13
4	Cellular Growth and Proliferation	1.26E-58 - 4.52E-13
5	Cell Death	1.34E-56 - 6.17E-13
6	Cellular Movement	1.45E-55 - 5.59E-13
7	Gene Expression	2.32E-49 - 6.23E-13
8	Tissue Development	7.7E-49 - 7.19E-13
9	Gastrointestinal Disease	3.24E-45 - 2.6E-13
10	Organismal Development	5.38E-45 - 7.19E-13

The predicted targets (from TargetScan) were examined by Ingenuity Pathway Analysis. Ranks were determined by right-tailed Fisher's Exact Test and were corrected by Benjamini-Hochberg Multiple Testing Correction.

B-H *P*-value  =  Benjamini-Hochberg *P*-value.

**Figure 4:**
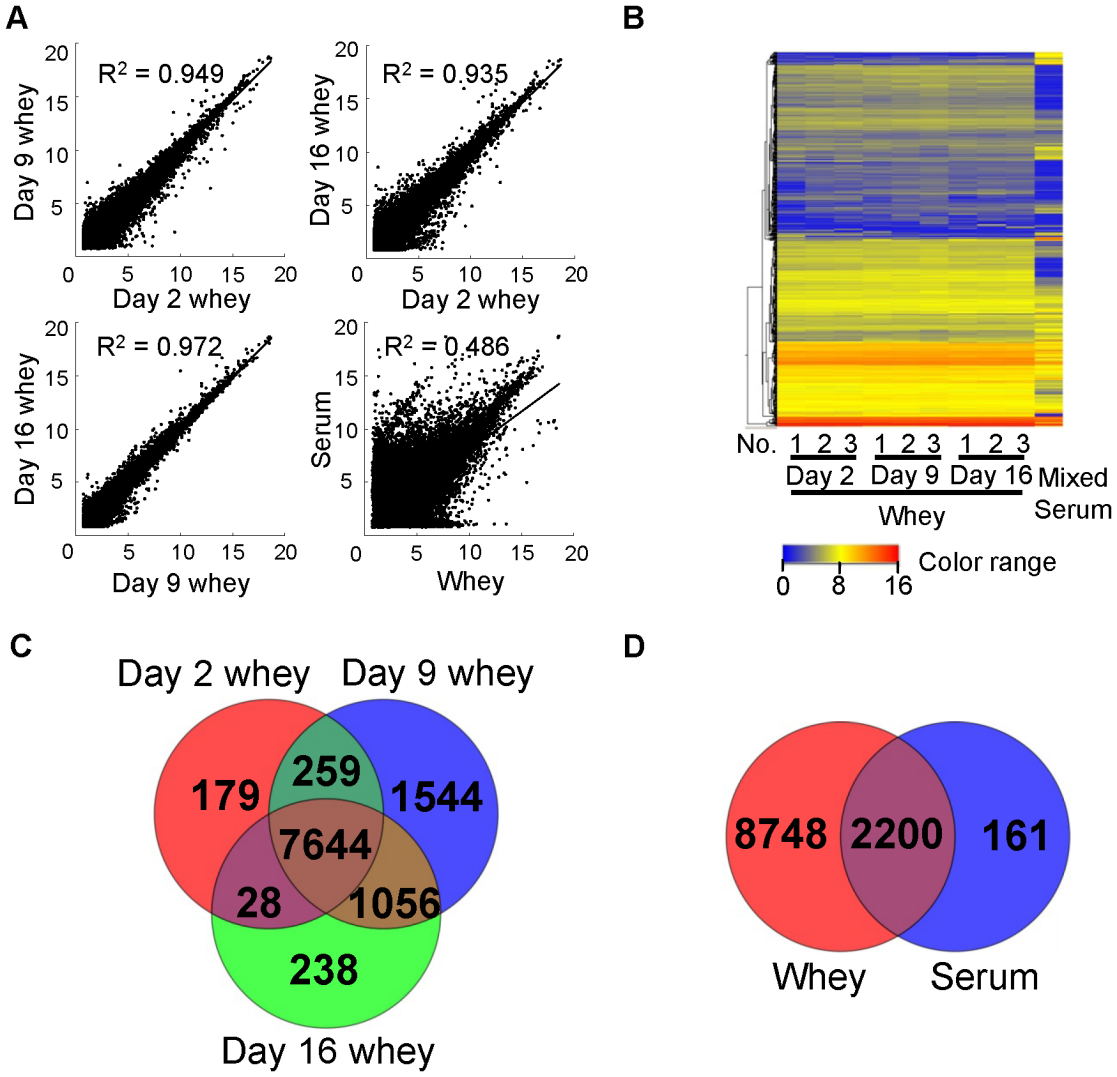
mRNA microarray profiles of rat whey and serum. (A) Scatter plots of averaged whey array data and mixed serum array data. (B) Heat map of the normalized array data for mRNAs detected in whey or mixed serum samples. (C) Venn diagram of numbers of transcripts detected in each type of whey. (D) Venn diagram of numbers of transcripts detected in whey and serum. Detailed signal intensities in heat map and mRNA transcript species in Venn diagram are shown in Table S4.

We previously detected some mRNAs in bovine whey [4], [5], and another study also showed that mRNAs exist in human whey [19]. Moreover, transcriptomic analyses of milk fat globules [10] and milk somatic cells [14] have been reported. However, there has been no comprehensive study of whey mRNA. Thus, we also performed mRNA microarray analysis of rat whey. To our knowledge, this is the first report of a comprehensive analysis of whey mRNAs. A scatter plot analysis (Fig. 4A) of mRNA microarray data obtained using the same amount of total RNA (100 ng; in the case of serum samples, total RNA was obtained by mixing RNA from three samples) showed that there are large differences between whey and serum, and few differences between whey collected from different postpartum stages in terms of mRNA expression patterns. The microarrays we used for mRNA analysis can detect 41,090 transcripts. We detected 10,948 transcripts in whey. A total of 8,110 transcripts were detected in d 2 whey, 10,503 in d 9 whey, and 8,966 in d 16 whey (Fig. 4B–D). Only 2,361 transcripts were detected in serum (Fig. 4B, D). A total of 2,200 transcripts were common to both whey and serum (Fig. 4D), but their signal intensities were quite different (Fig. 4B). The roles of the 10,948 transcripts detected in whey were analyzed by IPA. The detected transcripts were suggested to have roles in “RNA post-transcriptional modification”, “protein synthesis”, and “infectious disease” (Table 2). Comparison of the predicted targets of miRNAs and mRNA transcripts in whey revealed that some roles are shared such as “cell cycle” and “cell death”, but others are quite different from each other (Tables 1 and 2).

**Table 2:** Functions of transcripts detected in whey.

Rank	Category	B-H *P*-value
1	RNA Post-Transcriptional Modification	1.3E-31 - 3.49E-01
2	Protein Synthesis	8.01E-22 - 3.47E-01
3	Infectious Disease	8.01E-22 - 3.47E-01
4	Cell Death	7.85E-18 - 3.49E-01
5	Molecular Transport	4.79E-17 - 2.97E-01
6	Protein Trafficking	4.79E-17 - 2.97E-01
7	Cell Cycle	3.43E-16 - 3.49E-01
8	DNA Replication, Recombination, and Repair	2.18E-15 - 3.49E-01
9	Post- Translational Modification	4.38E-14 - 3.49E-01
10	Organismal Injury and Abnormalities	2.02E-13 - 3.14E-01

Transcripts detected by microarray analysis were examined by Ingenuity Pathway Analysis. Ranks were determined by the right-tailed Fisher's Exact Test and were corrected by Benjamini-Hochberg Multiple Testing Correction.

B-H *P*-value  =  Benjamini-Hochberg *P*-value.

**Figure 5:**
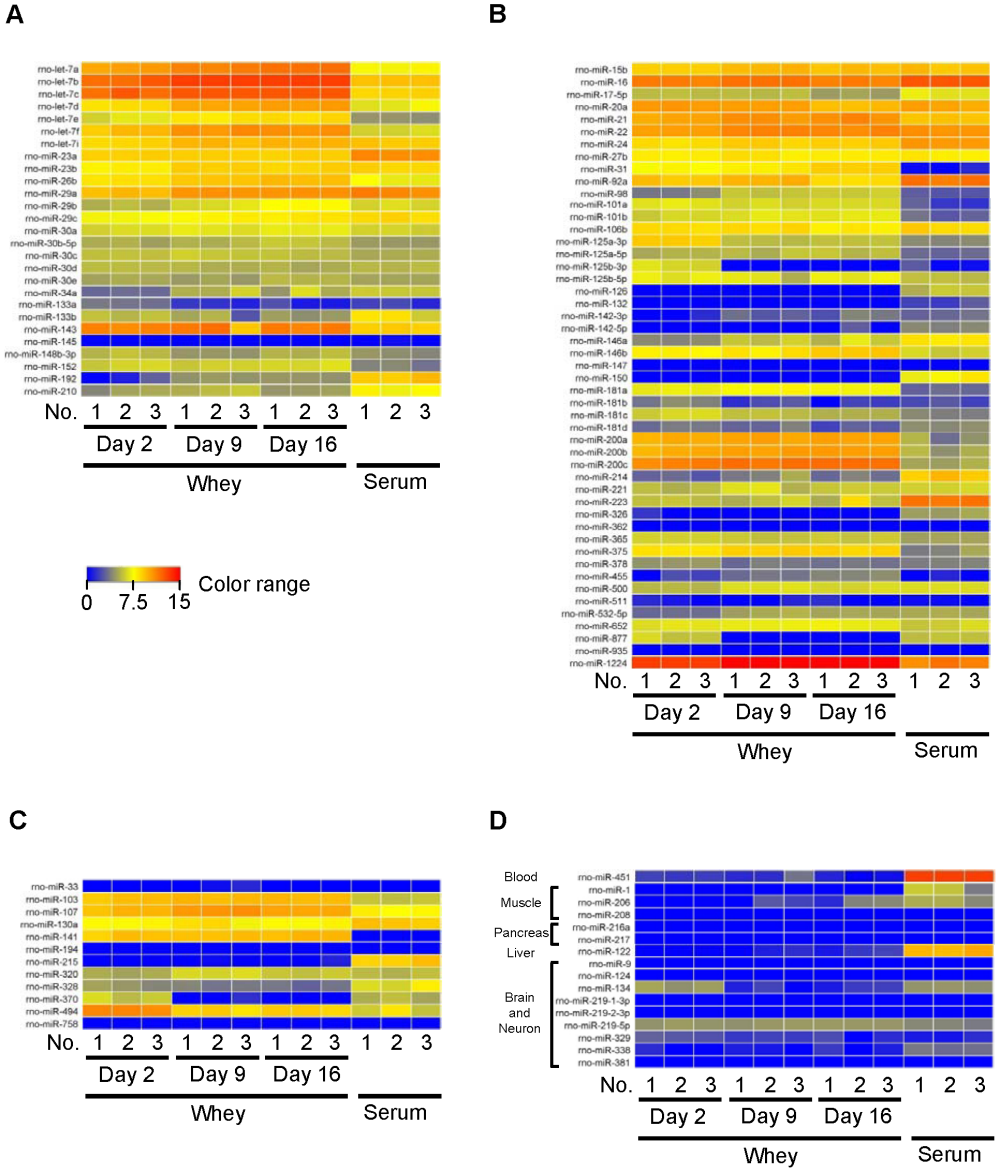
Heat maps of selected miRNAs from microarray data (detected or not). (A) Immune- and development-related miRNAs. (B) Immune-related miRNAs. (C) Development-related miRNAs. (D) Tissue-specific miRNAs. Detailed signal intensities are shown in Table S5.

**Figure 6:**
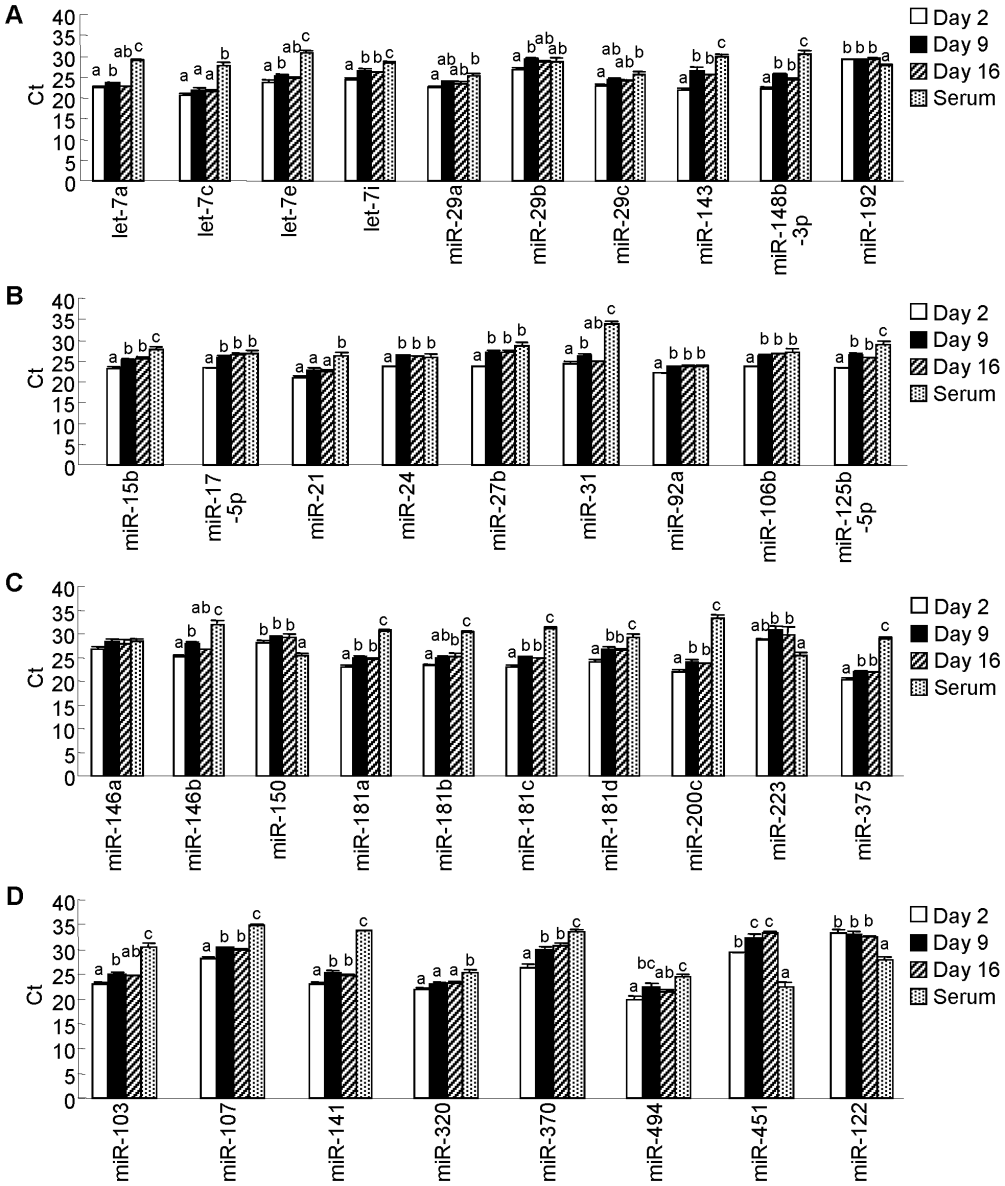
Quantitative PCR analysis of selected miRNAs in equal volumes of whey and serum. An aliquot of total RNA from 0.5-µL was used for each miRNA. Values are the mean ± SEM (n = 3). Means without letters in common differ (*P*<0.05, Tukey-Kramer HSD test).

Milk is considered to carry out its roles in neonatal physiological and immunological development. Thus, we selected immune- and development-related miRNAs with experimentally confirmed roles based on the annotation of the Pathway Central database (SABiosciences, Frederick, MD) and several previous studies [21]–[36], and created heat maps to visualize these miRNAs (regardless of whether or not they were detected) (Fig. 5A, both immune- and development-related roles; Fig. 5B, immune-related roles; Fig. 5C, development-related roles; Fig. 5D, tissue-specific miRNAs) (tissue-specific miRNAs are referred to in the following references: [6], [7], [13], [32], [37], [38]). Moreover, we further selected relatively well-studied miRNAs from heat maps and analyzed them by qPCR in the same volume of sample (Fig. 6A, both immune- and development-related miRNAs; Fig. 6B and C, immune-related miRNAs; Fig. 6D, metabolism-related and tissue-specific miRNAs). Many immune- and development-related miRNAs were detected and showed various expression levels; however, not all of them were detected; indeed, and most tissue-specific miRNAs were not detected (Fig. 5). Tissue-specific miRNAs (miR-451 and miR-122) were only detected in whey at very low levels by qPCR (Fig. 6D). These results are in accordance with those of milk from other species, such as humans [6], [13], cows [4], [7], and pigs [8], and suggest that milk whey miRNAs may have specific immune and development roles in neonates. Comparison of whey and serum qPCR analyses using the same volumes of samples showed that only miR-192, miR-150, and miR-223 (apart from the tissue-specific miRNAs miR-451 and miR-122) were detected at higher levels in serum than in whey. Our previous human milk whey study also showed that miR-150 and miR-223 were present at higher levels in serum than in whey [6]. Comparison of whey qPCR analyses using the same volumes of samples showed that levels of some miRNAs, such as let-7c, miR-29a, miR-29c, miR-192, miR-21, miR-146a, miR-150, miR-223, and miR-320, did not change during the lactation period (Fig. 6). On the other hand, other miRNAs such as, let-7i, miR-143, miR-148b-3p, miR-15b, miR-17-5p, miR-24, miR-27b, miR-92a, miR-106b, miR-125b-5p, miR-181a, miR-181c, miR-181d, miR-200c, miR-375, miR-107, miR-141, and miR-370, were present at higher levels in colostrum whey than in mature milk whey (Fig. 6). The results for miR-15b, miR-27b, and miR-106b are in accordance with those for bovine [4]. On the other hand, the levels of expression in colostrum whey and mature milk whey of miR-29b, and miR-223 are different from those of bovines [4]. That is to say, in comparison between colostrum and mature milk whey by qPCR in bovine, there was no significantly difference in miR-29b between colostrum and mature milk, and miR-223 was significantly higher in colostrum whey than in mature milk whey. However, in rat, miR-29b was significantly higher in d 9 milk whey than in d 2 colostrum whey, and there was no difference in miR-223 in colostrum whey and in mature milk whey. These results suggest that roles of whey miRNAs are common to several species while others are species-dependent. Among miRNAs that were present at higher levels in colostrum whey, let-7i, miR-148b-3p, miR-27b, and miR-125b-3p affect the function of antigen-presenting cells, and miR-15b, miR-24, miR-92a, miR-181a, miR-181c, and miR-181d affect T cell development and function [29], [30], [35]. Human [39] and bovine [40] colostrum has been reported to show anti-inflammatory effects. The mechanisms underlying these effects could be related to miRNAs detected at higher levels in colostrum whey. Because, for example, let-7i and miR-146b has been reported to down regulate TLRs [30], [35], and miR-125b has been reported to suppress TNF-α [30]. In the context of development, higher detected levels of miRNA in colostrum whey are interesting because miR-143, miR-148b-3p, and miR-141 are known to regulate intestinal function [41], [42] and miR-107 and miR-370 are known to modulate carbohydrate and lipid metabolism [27], [33].

**Figure 7:**
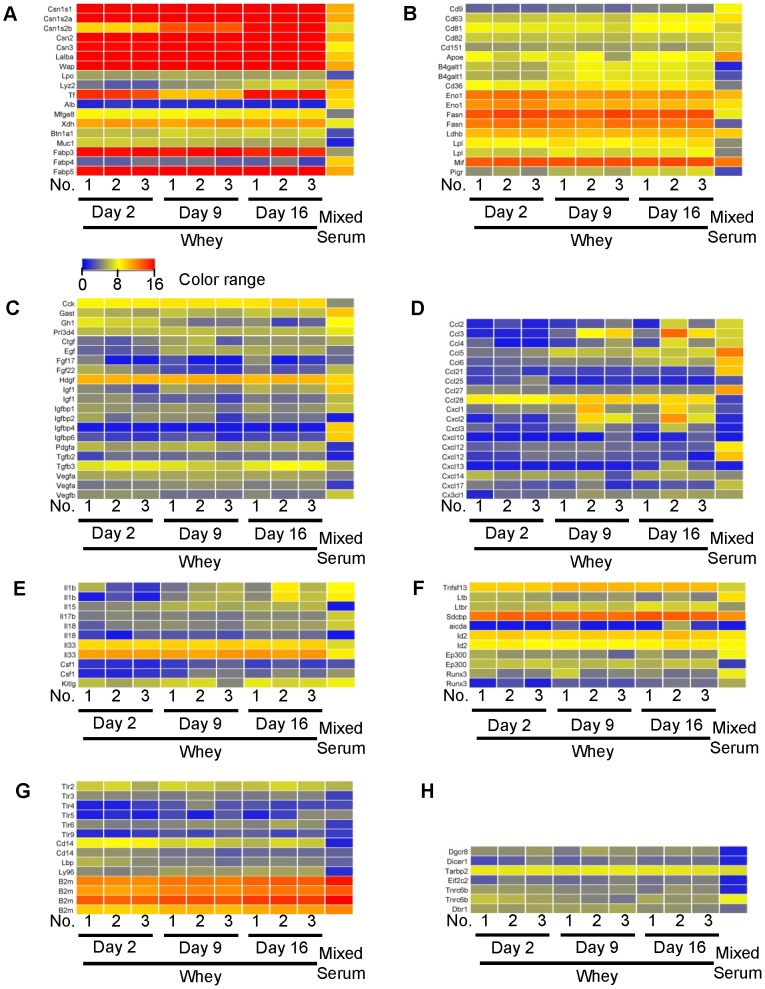
Heat maps of selected mRNA transcripts from microarray data (detected). (A) Milk protein transcripts. (B) Milk-derived exosome-related transcripts. (C) Hormone and growth factor transcripts (D) Chemokine transcripts. (E) Cytokine transcripts. (F) IgA induction-related transcripts (G) Pattern recognition receptor-related transcripts. (H) miRNA processing-related transcripts. Detailed signal intensities are shown in Table S6.

**Figure 8:**
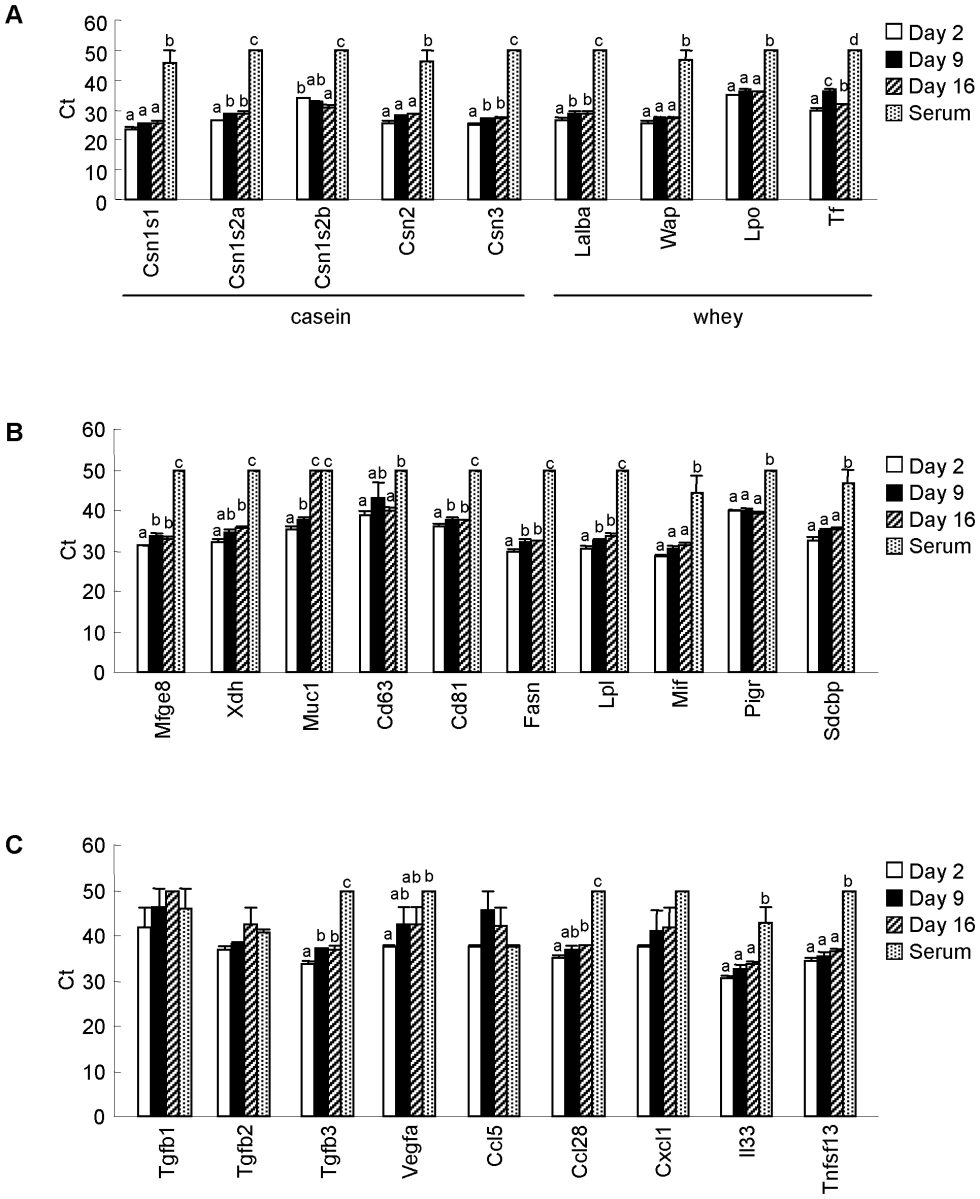
Quantitative PCR analysis of selected mRNAs in equal volumes of whey and serum. An aliquot of total RNA from 0.5-µL was used for each mRNA. Non-detected mRNAs were assigned a Ct value of 50. Values are the mean ± SEM (n = 3). Means without letters in common differ (*P*<0.05, Tukey-Kramer HSD test).

According to our previous study [4], mRNAs in milk whey were thought to originate from milk-derived exosomes. Exosomes are small (10–100 nm diameter) membrane vesicles present in many body fluids [43], including milk whey [6], [8], [9], [13], [39], [43]. And a previous report showed that exosomes contain not only miRNA, but also mRNA [44]. In addition to being nutritious, milk may act as an immune enhancer and may have anti-microbial properties [2], [3]. Thus, we selected mainly immune-, development- and exosome-related transcripts (i.e., milk protein, milk-derived exosome-related [39], hormone and growth factor, chemokine, cytokine, IgA induction-related [45]–[47], pattern recognition receptor-related, and miRNA processing-related transcripts [48]–[50]) from our mRNA microarray data (limited to detected transcripts) and created heat maps. These are shown in Figure 7 (Fig. 7A, milk protein; Fig. 7B, milk-derived exosome-related; Fig. 7C, hormone and growth factor; Fig. 7D, chemokine; Fig. 7E, cytokine; Fig. 7F, IgA induction-related; Fig. 7G, pattern recognition receptor-related; Fig. 7H, miRNA processing-related). Moreover, we selected some mRNAs from the heat maps and analyzed them by qPCR in the same volumes of samples (Fig. 8). Milk protein and milk-derived exosome-related transcripts were relatively abundant (Fig. 7). In other categories, *Sdcbp* and *B2m* were detected at relatively high levels. These transcripts encode proteins that have been detected in milk-derived exosomes [39]. Thus, these transcripts can be also categorized as milk-derived exosome-related transcripts. These results strongly suggest that transcripts detected in whey may be derived from milk-derived exosomes. Comparison of whey and serum qPCR analyses using the same volumes of samples showed that almost all selected milk protein-related mRNAs and milk-derived exosome-related mRNAs were present at significantly higher levels in whey than in serum except for *Muc 1* and *Cd63* (*Muc 1* was detected at the same level as in d 16 whey, and *Cd63* was detected at the same level as in d 9 whey). However, some growth factor- and chemokine-related mRNAs such as *Tgfb1*, *Tgfb2*, *Ccl5*, and *Cxcl1* mRNAs, were detected at similar levels in whey and serum. Comparison of whey qPCR analyses using the same volumes of samples, levels of some mRNAs, such as *Csn1s1*, *Csn2*, *Wap*, *Lpo*, *Cd63*, *Mif*, *Pigr*, *Sdcbp*, *Tgfb1*, *Tgfb2*, *Vegfa*, *Ccl5*, *Cxcl1*, *Il33*, and *Tnfsf13*, did not change during the lactation period (Fig. 8). In contrast, other mRNAs such as *Csn1s2a*, *Csn3*, *Lalba*, *Tf*, *Mfge8*, *Muc1*, *Cd81*, *Fasn*, *Lpl*, and *Tgfb3*, were detected at significantly higher levels in colostrum whey than in mature milk whey (Fig. 8). Among these transcripts, *Tf* and *Csn1s2b* showed different expression patterns compared to other transcripts. Whey *Tf* levels decreased from d 2 to d 9, and increased from d 9 to d 16. This result is in accordance with a previous report of transferrin protein in rat milk and mRNA in the rat mammary gland [51]. *Csn1s2b* was the only transcript detected at significantly higher levels in d 16 whey than in colostrum whey. Although *Csn1s1*, *Csn1s2a*, *Csn1s2b*, *Csn2*, and *Csn3* are casein transcripts that encode different types of casein proteins, these transcripts showed differential expression patterns during lactation. Previous reports have shown that casein mRNAs are expressed in monocytes, monocytic cell lines, T cell, and cytotoxic T-cell lines, and have suggested that they may have immunomodulatory functions [52], [53]. Each casein gene showed a different expression pattern during lactation, which suggests that whey mRNAs may have some physiological roles in neonates and that their roles may differ according to lactation stage.

We detected 10,948 transcripts in rat milk whey. Maningat et al. [10] detected 14,070 transcripts in human milk fat globules. They listed milk protein genes (*CSN1S1*, *CSN2*, *CSN3*, *LALBA*, *XDH*, *MFGE8*, *MUC1*), milk-derived exosome-related protein genes (*FASN*, *CD81*, and *CD36*), and other genes (e.g., *TPT1*, *SPP1*, *CEL*, *GNB2L1*, *TMSB4X*, and so forth) among the most highly expressed genes in human milk fat globules. Among them, high expression of milk protein and milk-derived exosome-related transcripts are in accordance with our results, but the expression levels of other transcripts, such as *TPT1*, *SPP1*, *CEL*, *GNB2L1*, and *TMSB4X*, are not. These transcripts were absent or present at very low levels in our study. Wickramasinghe et al. [14] detected 19,094 transcripts in bovine milk somatic cells by next-generation sequencing. They listed milk protein genes (*CSN1S1*, *CSN1S2*, *CSN2*, *CSN3*, *LALBA*, and *LGB*) and other genes (e.g. *GLYCAM1* and *SPP1*) among those highly expressed in bovine milk somatic cells; however, they identified only the *APOE* gene among milk exosome-related protein genes. Some roles of mRNAs may be common to milk whey, milk fat globules, and milk cells, while others may be different or roles of milk mRNA might be different between species.

**Figure 9:**
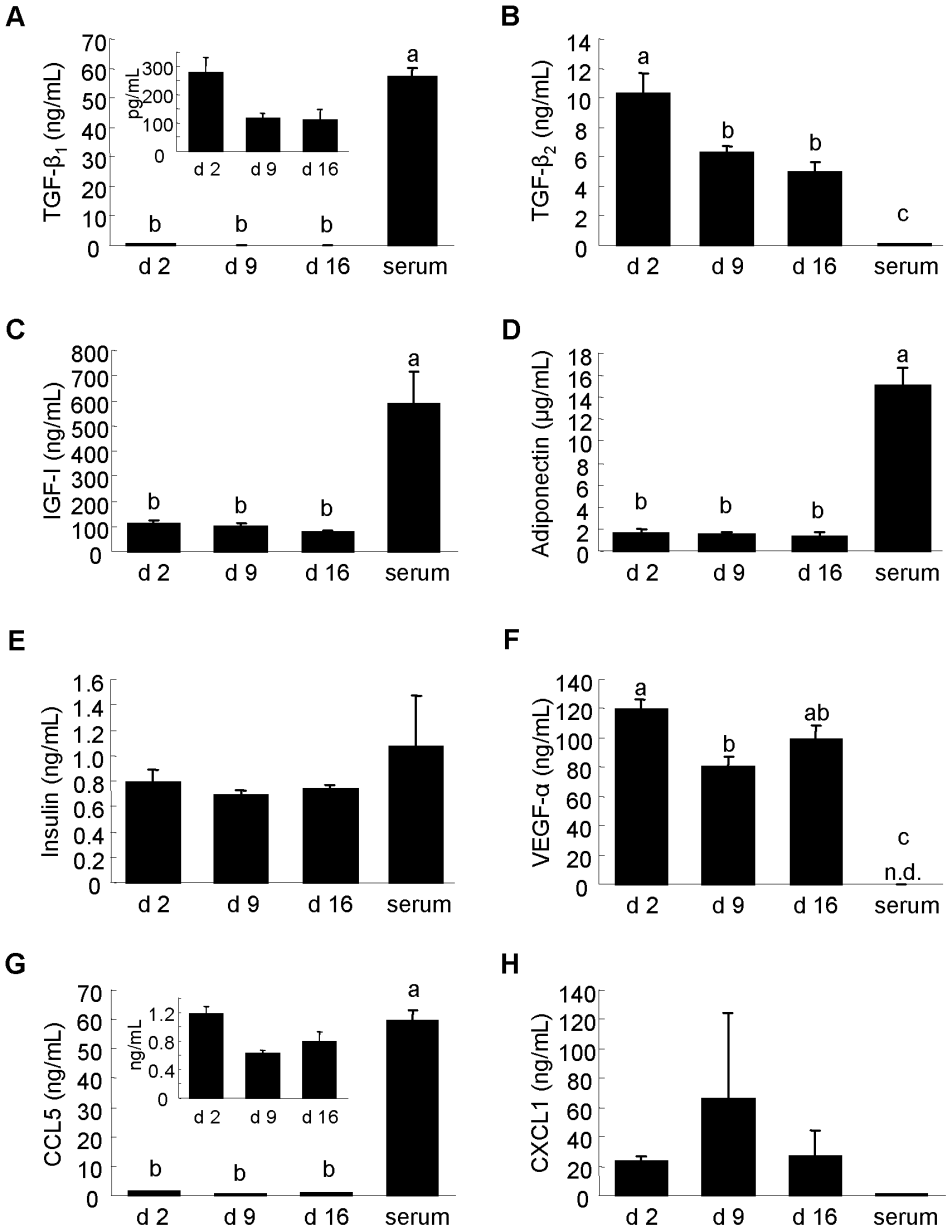
Growth factor, hormone and chemokine concentrations in whey and serum. (A) Transforming growth factor (TGF)-β_1_. (B) TGF-β_2_. (C) IGF-I. (D) Adiponectin. (E) Insulin. (F) VEGF-α. (G) CCL5. (H) CXCL1. (A-D) Measured using ELISA kits. (E-H) Measured using Milliplex panels. Values are the mean ± SEM (n = 3). Means without letters in common differ (*P*<0.05, Tukey-Kramer HSD test).

Many growth factors, hormones, and chemokines are present in milk [1]–[3]. Hence, we also measured the concentrations of these in whey and serum and compared them to the corresponding mRNA results. TGF-β_1_, IGF-I, adiponectin, and CCL5 concentrations were significantly higher in serum than in whey (Fig. 9A, C, D, G). In contrast, TGF-β_2_ and VEGF-α concentrations were significantly higher in whey than in serum (Fig. 9B, F). Comparing different whey samples, the TGF-β_2_ concentration was higher in colostrum whey than in mature milk whey (d 9 and d 16), while that of VEGF-α was higher in colostrum whey than in d 9 mature milk whey (Fig. 9B, F). These results show that levels of protein factors in whey are quite different from those in serum, and suggest that neonatal requirements for these proteins could differ according to developmental stage. The mRNA results (Fig. 8C) did not reflect these protein results. Interestingly, although some hormones were not detected or were detected at very low levels at the mRNA level, their protein levels were relatively high. Although adiponectin and insulin were considered “not detected” by the microarray (raw signal<20) and qPCR analyses, and IGF-I was detected at low levels using microarray (Fig. 7C), adiponectin protein was present at µg/mL levels and insulin and IGF-I at ng/mL levels in whey and serum (Fig. 9C–E). These biological factors have also been detected in whey from other species and have been reported to show biological activities in the neonatal intestine [1], . Our findings that mRNA expression level does not correlate with protein level in whey and serum shows that mRNAs in whey and serum are not only debris remaining after translation.

Recently, Wang et al. reported that food-derived and microbiota-derived miRNAs are present in human blood [56], and Gu et al. reported that the blood of piglets fed colostrum only contains higher levels of miRNAs present at high levels in colostrum than the blood of piglets fed mature milk only [8]. We previously reported that miRNA in milk and milk protein mRNA in milk are resistant to acidic conditions and to RNase [4]. According to these results, miRNAs and mRNAs in milk could function in the neonatal gastrointestinal tract. Many biological factors in milk have been described [1]–[3] and we confirmed the presence of some of them in this study. These biological factors have been reported to exert biological effects on the neonatal intestine [1], [54], [55]. For example, adiponectin may regulate some miRNAs *in vivo* [57]. Biological factors such as proteins, miRNAs, and mRNAs could be affected by each other.

In conclusion, we demonstrated that miRNAs are present in rat milk whey, as they are in milk whey in other mammalian species such as humans, cows, and pigs, and that mRNA are also present in rat milk whey. We also showed that the concentration of RNA in colostrum whey is much higher than that in mature milk whey. Moreover, our microarray and qPCR results showed that the expression levels of RNA in whey change during lactation. We further showed that mRNA expression levels in milk do not correlate with protein levels in whey. Over the past few years, circulating RNAs have received much attention. However, most studies of circulating RNAs have focused on their function as diagnostic biomarkers, rather than as biological factors [58]. In this study, we did not show that RNAs in whey could function *in vivo*. Therefore, further studies are clearly needed. However, before investigating the roles of milk RNAs, it is important to know the contents of functional RNAs. There have been reports that functional RNAs, especially circulating miRNAs, exist in different forms, for example packaged in exosomes [19], [43], [58], microvesicles [58], and HDL [59], and as complexes with RNA-binding proteins [60], and that RNAs can be transferred into cells. Moreover, there have been reported that milk fat fraction also contains RNA [10], [11], and pre-miRNA (60–80 nt) might be contained in milk whey according to our result (Fig. 1). How many forms of milk whey RNAs exist? What are the proportions of those forms in the distribution of RNA? Additionally, Munch et al. [11] very recently reported that levels of several miRNAs in the human milk lipid fraction were altered by the maternal diet. Our previous studies [6] showed clear individual specificities in human breast milk whey miRNA expression patterns. Which factors affect RNA expression patterns in milk? Many aspects of milk RNAs remain to be resolved. However, our results will aid future *in vivo* studies of milk RNAs using experimental animals such as rats.

## Supporting Information

Table S1 **Primers used for miRNA assays.**
(DOC)

Table S2 **TaqMan probes used for mRNA assays.**
(DOC)

Table S3 **Expressed miRNA species and their signal intensities.**
(XLS)

Table S4 **Expressed mRNA transcripts and their signal intensities.**
(XLS)

Table S5 **miRNA signal intensities in heat maps.**
(XLS)

Table S6 **mRNA transcript signal intensities in heat maps.**
(XLS)
