# Supplementary material for: Time-Dependent Expression Profiles of microRNAs and mRNAs in Rat Milk Whey
Source: PLoS One. 2014 Feb 12;9(2):e88843. doi: 10.1371/journal.pone.0088843 (PMC3923055; doi:10.1371/journal.pone.0088843)
Supplement: Table S1 — Primers used for miRNA assays. (DOC) [file pone.0088843.s001.doc]

**TABLE S1.** Primers used for miRNA assays.

|  | miRNA | Product no. |
| --- | --- | --- |
| 1 | let-7a | MS00005460 |
| 2 | let-7c | MS00005467 |
| 3 | let-7e | MS00012922 |
| 4 | let-7i | MS00000028 |
| 5 | miR-29a | MS00000168 |
| 6 | miR-29b | MS00005544 |
| 7 | miR-29c | MS00000175 |
| 8 | miR-143 | MS00000420 |
| 9 | miR-148b-3p | MS00000448 |
| 10 | miR-192 | MS00000539 |
| 11 | miR-15b | MS00013097 |
| 12 | miR-17-5p | MS00013118 |
| 13 | miR-21 | MS00013216 |
| 14 | miR-24 | MS00005537 |
| 15 | miR-27b | MS00000154 |
| 16 | miR-31 | MS00000203 |
| 17 | miR-92a | MS00005579 |
| 18 | miR-106b | MS00000301 |
| 19 | miR-125b-5p | MS00005600 |
| 20 | miR-146a | MS00000441 |
| 21 | miR-146b | MS00013062 |
| 22 | miR-150 | MS00000455 |
| 23 | miR-181a | MS00013125 |
| 24 | miR-181b | MS00005677 |
| 25 | miR-181c | MS00013132 |
| 26 | miR-181d | MS00013139 |
| 27 | miR-200c | MS00000595 |
| 28 | miR-223 | MS00033320 |
| 29 | miR-375 | MS00033516 |
| 30 | miR-103 | MS00005586 |
| 31 | miR-107 | MS00033173 |
| 32 | miR-141 | MS00000413 |
| 33 | miR-320 | MS00013433 |
| 34 | miR-370 | MS00037457 |
| 35 | miR-494 | MS00028952 |
| 36 | miR-122 | MS00000315 |
| 37 | miR-451 | MS00001113 |
