# Supplementary material for: Time-Dependent Expression Profiles of microRNAs and mRNAs in Rat Milk Whey
Source: PLoS One. 2014 Feb 12;9(2):e88843. doi: 10.1371/journal.pone.0088843 (PMC3923055; doi:10.1371/journal.pone.0088843)
Supplement: Table S2 — TaqMan probes used for mRNA assays. (DOC) [file pone.0088843.s002.doc]

**TABLE S2.** TaqMan probes used for the mRNA assays

|  | mRNA | Product no. |
| --- | --- | --- |
| 1 | Csn1s1 | Rn01524597_m1 |
| 2 | Csn1s2a | Rn01524579_m1 |
| 3 | Csn1s2b | Rn00597435_m1 |
| 4 | Csn2 | Rn01524626_m1 |
| 5 | Csn3 | Rn00580733_m1 |
| 6 | Lalba | Rn01532920_m1 |
| 7 | Wap | Rn01461679_m1 |
| 8 | Lpo | Rn01751775_m1 |
| 9 | Tf | Rn01445482_m1 |
| 10 | Mfge8 | Rn00563082_m1 |
| 11 | Xdh | Rn00567654_m1 |
| 12 | Muc1 | Rn01462585_m1 |
| 13 | Cd63 | Rn00567481_m1 |
| 14 | Cd81 | Rn00565272_m1 |
| 15 | Fasn | Rn00569117_m1 |
| 16 | Lpl | Rn00561482_m1 |
| 17 | Mif | Rn00821234_g1 |
| 18 | Pigr | Rn00562362_m1 |
| 19 | Sdcbp | Rn01220868_m1 |
| 20 | Tgfb1 | Rn00572010_m1 |
| 21 | Tgfb2 | Rn00676060_m1 |
| 22 | Tgfb3 | Rn00565937_m1 |
| 23 | Vegfa | Rn00582935_m1 |
| 24 | Ccl5 | Rn00579590_m1 |
| 25 | Ccl28 | Rn00586715_m1 |
| 26 | Cxcl1 | Rn00578225_m1 |
| 27 | Il33 | Rn01759837_m1 |
| 28 | Tnfsf13 | Rn01467490_g1 |
| 29 | Acdc | Rn00595250_m1 |
| 30 | Insulin 1 | Rn02121433_g1 |
| 31 | Tnfrsf11b | Rn00563499_m1 |
